# Optimizing PCR Detection of Zika Virus from Various Body Fluids

**DOI:** 10.4269/ajtmh.18-0755

**Published:** 2018-12-17

**Authors:** Rodion Gorchakov, Rebecca M. Berry, Shital M. Patel, Hana M. El Sahly, Shannon E. Ronca, Kristy O. Murray

**Affiliations:** 1 Department of Pediatrics-Tropical Medicine, Baylor College of Medicine and Texas Children’s Hospital, Houston, Texas;; 2 Department of Medicine, Baylor College of Medicine, Houston, Texas;; 3 Department of Molecular Virology and Microbiology, Baylor College of Medicine, Houston, Texas

## Abstract

Current diagnostic protocols of acute Zika virus (ZIKV) infection focus on detection of viral RNA in serum or urine using reverse transcription quantitative polymerase chain reaction (RT-qPCR); however, detecting infection can be a challenge, given that 80% of people with acute ZIKV infection are asymptomatic, and the window to detect viremia in serum is short. The ability to extend that window is needed to detect ZIKV at later time points after infection, particularly in high-risk individuals such as pregnant women. We evaluated RNA extraction methods to optimize detection of ZIKV in various body fluids using RT-qPCR as a means of improving the analytical sensitivity of detection. We optimized methods for ZIKV RNA recovery from a number of body fluids by spiking with three varying concentrations of virus, then comparing recovery with that of spiked buffer control. RNA extraction protocols were adjusted as necessary for maximum RNA recovery. Adjustment of the elution step was essential for improved ZIKV RNA recovery from whole blood, saliva, vaginal secretions, and breast milk. Optimal recovery from urine samples required the addition of Urine Conditioning Buffer, and the use of RLT Plus buffer and RNeasy Mini Spin Columns was necessary for RNA extractions from semen samples. Optimized QIAamp MinElute Virus Spin Kit (QIAGEN, Valencia, CA) protocol followed by the singleplex ZIKV RT-qPCR assay provided a reliable method for detection of ZIKV RNA in a variety of biological samples. Improved diagnostics are crucial for timely detection and diagnosis, particularly during pregnancy when the consequences of ZIKV infection can greatly impact the developing fetus.

## INTRODUCTION

Zika virus (ZIKV), a member of *Flavivirus* genus, emerged in the Americas in 2015 and is a significant public health concern.^1–3^ The virus has been known to circulate with limited reported activity in the Old World since before the middle of the twentieth century.^2,3^ It first appeared in the continental New World in Brazil in March 2015, initiating an explosive outbreak that resulted in millions of infections throughout South, Central, and North America, including the U.S. territory of Puerto Rico, within 1 year.^1–3^ Most of the clinical cases reported in the U.S. states have been associated with travel to the affected areas.^4^ Since 2015, more than 5,000 such travel-associated cases have been documented, mostly in 2016. The number of locally acquired ZIKV cases in the United States is about 20 times lower than travel-associated cases, with limited autochthonous transmission confined to Florida and south Texas.^4^

Zika virus is mosquito-borne, transmitted to humans by a bite of *Aedes* spp. mosquitoes.^2,3^ Humans develop a level of viremia high enough to propagate the transmission cycle.^1–3^ Other routes of ZIKV transmission in humans have been documented, including sexual, congenital, breast milk, and blood transfusion.^1–3,5–8^ Transmission through organ transplantation is a possibility for ZIKV as it was reported for another *flavivirus*, West Nile virus (WNV).^9^

About 80% of infections with ZIKV are asymptomatic.^3^ Clinical manifestations of ZIKV infection are typically mild with acute onset of fever with maculopapular rash, arthralgia, conjunctivitis, myalgia, and headache, lasting from several days to 2 weeks.^1–3^ In rare cases, ZIKV infection can result in serious complications, including Guillain– Barré syndrome.^2,3^ The most severe ZIKV infection complications occur during pregnancy, leading to severe birth defects, death, and poor clinical outcomes in the newborn, with the condition collectively known as congenital Zika syndrome.^3,10^ The U.S. Zika Pregnancy Registry reported ZIKV-associated birth defects, including congenital microcephaly, in up to 5% of neonates born to mothers with possible recent ZIKV infection during pregnancy.^10,11^

Zika disease onset usually coincides with viremia and viruria.^2,3^ Therefore, nucleic acid tests on paired serum and urine samples are recommended for the laboratory diagnosis of acute ZIKV infection.^2^ Detection of viral RNA in sera or urine provides conclusive diagnosis of infection, but the timeframe for it is limited to 1–2 weeks post onset of symptoms because of fast decline of virus presence in serum and urine.^2,3^ Tests for ZIKV-specific IgM antibodies in serum are also used as a diagnostic evidence for ZIKV infection and might increase the window for diagnosis to several months, but ZIKV serology is complicated by considerable cross-reactivity with other related flaviviruses, particularly in dengue-endemic areas.^2^ Considering these diagnostic constraints, it would be ideal to optimize the ability to detect RNA for a longer window of time, particularly in high-risk individuals (i.e., pregnant women or their sexual partners), or individuals with serious complications such as Guillain–Barré syndrome. We recently reported our laboratory’s experience in detecting ZIKV RNA in saliva and whole blood up to 14 and 81 days post onset of symptoms in one acute ZIKV case, respectively.^12^ Given these findings, the purpose of this study was to identify viral RNA extraction methods that maximized ZIKV RNA recovery from a given sample type to further strengthen diagnostic protocols for detection of ZIKV in various body fluids.

## MATERIALS AND METHODS

### Human and viral samples.

Collection of clinical specimens was performed by standard procedures under a research protocol enrolling suspected or confirmed cases of ZIKV infection in U.S. residents. The studies were approved by Baylor College of Medicine Institutional Review Board, protocols H-30533 and H-39865. Serum specimens were obtained by collection and processing of venous blood in BD SST vacutainers (BD, Franklin Lakes, NJ). Whole blood specimens were obtained by collection and processing of venous blood in BD K2EDTA vacutainers (BD). As it is shown that ZIKV remains associated with red blood cell fraction of the whole blood,^12^ plasma was separated and discarded, and the remaining blood cells and a small portion of plasma were mixed. Analogous to the setup and description from a previous WNV study,^13^ this fraction is referred to as whole blood from this point forward, as it contains all major components of whole blood: erythrocytes, leukocytes, thrombocytes, and a portion of plasma. Urine specimens were spun at 1,000 × *g* and the supernatant was collected for testing. Saliva swabs and vaginal secretions swabs were obtained with BD BBL CultureSwab Sterile Single Swab (BD), which is free of media or preservatives. Semen specimens were collected in a sterile urine cup, allowed for liquefaction, and used without separation. Breast milk specimens were collected using the subject’s own breast pump, then transferred to a sterile specimen cup.

Zika virus–positive control, strain MEX I-7, was provided by University of Texas Medical Branch World Reference Center for Emerging Viruses and Arboviruses. The strain was isolated in 2016 from mosquito samples collected in Mexico and passaged a total of five times on Vero cells.

### Spiking of clinical specimens.

Ten aliquots of each sample matrix were prepared, with 200 µL volume for liquid samples. AVE buffer, the elution buffer from the QIAamp MinElute Virus Spin Kit (QIAGEN, Valencia, CA), was used as the spiking reference control buffer to generate expected cycle threshold (Ct) values. The aliquots were mixed with 200 µL of the lysis buffer from the kit, which is AL buffer containing 28 ng/µL of carrier RNA. The aliquots were spiked in triplicates with 10 µL of viral cell culture supernatant containing three levels of viral load, and a single aliquot was left unspiked to confirm ZIKV-negative status of the samples from the donors. To obtain high, medium, and low spiking loads, the original viral cell culture supernatant was diluted in culture media so that the reference buffer samples’ final Ct values were in the range of 19–21, 27–29, and 33–35, respectively. Spiked samples were stored at −80°C before proceeding to RNA extraction.

#### Spiking of WB specimens.

To facilitate lysis of 200 µL of packed blood cells specimens, which approximate 400 µL of the original venous blood specimens, a total of 400 µL of AL along with 200 µL of phosphate buffered saline (PBS) were added to whole blood samples (Table 1). AL for whole blood specimens did not have carrier RNA as this matrix has sufficient amount of cellular nucleic acid.

**Table 1:** The original QIAamp MinElute Virus Spin Kit protocol and its optimized versions

Step*	Original protocol	Optimization A	Optimization B	Optimization C
N/A	No sample pretreatment	†	†	Add 70 µL of Urine Conditioning Buffer to 1 mL of sample, vortex, centrifuge at 3,000 × *g* for 15 minutes, discard supernatant
1	Pipet 25 μL of Protease into a tube	WB only—50 μL of Protease	50 μL of Protease	Resuspended pellet in a mix of 200 µL AVE, 200 µL AL/carrier RNA, and 25 µL Protease
2	Add 200 μL of sample	WB only—200 µL of packed blood cells and 200 µL of PBS	†	N/A
3	Add 200 μL of buffer AL/carrier RNA, vortex	WB only—400 μL of buffer AL	†	N/A
4	Incubate at 56°C for 15 minutes	†	30 minutes	†
N/A	N/A	†	Add 1.6 mL of buffer RLT Plus/1% βME, incubate at RT for 5 minutes	†
5	Briefly centrifuge the tube to remove drops from the inside of the lid	†	†	†
6	Add 250 μL of 100% ethanol, vortex. Incubate for 5 minutes at room temperature	WB only—500 µL of ethanol	1.4 mL of ethanol	†
7	Briefly centrifuge the tube to remove drops from the inside of the lid	†	†	†
8	Apply onto the column. Centrifuge at 21,100 × *g* for 1 minute. Place the column in a clean collection tube	Centrifuge at 900 × *g* for 1 minute, followed by 1 minute 21,100 × *g*	Apply onto the RNeasy Mini Spin Column. Centrifuge at 900 × *g* for 1 minute, followed by 1 minute 21,100 × *g*	†
9	Add 500 μL of buffer AW1 without wetting the rim. Centrifuge at 21,100 × *g* for 1 minute. Place the column in a clean collection tube	WB only – repeat step 9	†	†
10	Add 500 μL of buffer AW2 without wetting the rim. Centrifuge at 21,100 × *g* for 1 minute. Place the column in a clean collection tube	†	†	†
11	Add 500 μL of 100% ethanol without wetting the rim. Centrifuge at 21,100 × *g* for 1 minute. Place the column in a clean collection tube	†	†	†
12	Place the column in a clean collection tube. Centrifuge at 21,100 × *g* for 3 minutes	†	†	†
13	Place the column into a new collection tube, open the lid, and incubate the assembly at 56°C for 3 minutes	†	†	†
14	Place the column in a clean tube. Apply 30 of buffer AVE to the center of the membrane. Close the lid and incubate at room temperature for 1 minute. Centrifuge at 21,100 × *g* for 1 minute	Incubate at 56°C for 5 minutes	Incubate at 56°C for 5 minutes, centrifuge at 21,100 × *g* for 1 minute, reload eluate, incubate at 56°C for 1 minute, centrifuge at 21,100 × *g* for 1 minute	†

βME = β-mercaptoethanol; WB = whole blood. The modifications to the original protocol are indicated per optimization versions.

* Step number is according to the manufacturer’s original protocol.

† No modification made to the original protocol.

#### Spiking of swab specimens.

Saliva and vaginal secretions swabs were first incubated in 250 µL of AL/carrier RNA for 10 minutes at room temperature and were then compressed against the inside of the tube to expel the liquid before removing and discarding. Remaining volume of the lysis buffer was about 200 µL, and 200 µL of PBS was added to adjust the total sample volume according to the QIAGEN protocol.

### RNA extraction.

The baseline method of RNA extraction from the study specimens was the QIAamp MinElute Virus Spin Kit (QIAGEN) according to the manufacturer’s protocol^14^ with elution in 30 µL of AVE buffer. All centrifugations were performed at maximum speed (21,100 × *g* [14,800 rpm]), except where indicated below. Each extraction batch included positive (ZIKV MEX I-7 diluted supernatant) and negative (AVE) extraction controls. Depending on the sample type, three sets of adjustments to the manufacturer’s protocol (Optimization A through C) were shown to be beneficial for ZIKV RNA recovery. The details of the three optimizations are described in the following text and Table 1 provides the step-by-step workflow of the original and optimized protocols.

#### Optimization A.

Sample load on the column (Step 8 of the QIAGEN protocol, Table 1) was performed at 900 × *g* (3,000 rpm), followed by 1 minute centrifugation at maximum speed. Elution incubation (Step 14, Table 1) was performed at 56°C for 5 minutes. For WB specimens, only – 50 µL of Protease (Step 1, Table 1) and 500 µL of ethanol (Step 6, Table 1) were used proportionally to the lysis/sample volume; and one extra wash with AW1 wash buffer of the kit (Step 9, Table 1) was performed to achieve clear flowthrough.

#### Optimization B.

For spiked semen specimens, 50 µL of Protease was added, and the incubation was extended to 30 minutes. Then, additional lysis buffer (1.6 mL of RLT Plus buffer [QIAGEN] containing 1% β-mercaptoethanol) was added, and the sample was incubated at room temperature for 5 minutes. After adding 1.4 mL of ethanol, the sample was loaded onto the RNeasy Mini Spin Column (QIAGEN) at 900 × *g* (3,000 rpm), followed by 1 minute centrifugation at maximum speed. The column was washed and dried per QIAamp MinElute Virus Spin Kit protocol, and the elution incubation step (Step 14, Table 1) was performed at 56°C for 5 minutes, followed by elution centrifugation. The eluate was reloaded on the same column, incubated at 56°C for 1 minute, and centrifuged for final elution.

#### Optimization C.

For 1-mL aliquots of urine specimens, proportional volume (50 µL) of spiking material was added directly to freshly collected urine. Seventy microliters of Urine Conditioning Buffer (UCB; Zymo Research, Irvine, CA) was mixed in. Samples were centrifuged at 3,000 × *g* (5,600 rpm), and pellet was resuspended in a mix of 200 µL AVE, 200 µL AL/carrier RNA, and 25 µL Protease. The sample was further processed starting with Step 4 of the manufacturer’s protocol (Table 1). Two intermediate −80°C freezing steps were tested: 1) right after spiking (then UCB added while still frozen and thawed with constant mixing); and 2) freezing the UCB pellet.

#### Alternative optimizations.

Additional procedures and reagents/supplies were tested to improve viral RNA recovery from semen and urine samples, including buffers and columns of RNeasy Plus Mini Kit or QIAamp Viral RNA Mini Kit, QIAzol, QIAshredder columns, ATL buffer (all QIAGEN), Direct-zol RNA MiniPrep Kit (Zymo Research), RNasin Plus RNase Inhibitor (Promega, Madison, WI), SUPERase In RNase Inhibitor (Thermo Fisher Scientific, Waltham, MA), Protector RNase Inhibitor (Roche, Indianapolis, IN), ProtectRNA RNase Inhibitor (Sigma-Aldrich, St. Louis, MO), and Aptima Urine Specimen Transport Tubes (Hologic, Inc., Marlborough, MA). The summary of these experiments is presented in Supplemental Tables 1 and 2.

### Viral RNA detection.

Five microliters of extracted RNA were used in a duplicate 20-µL reaction with TaqMan Fast Virus 1-Step Master Mix (Thermo Fisher Scientific) on ViiA 7 Real-Time PCR System (Thermo Fisher Scientific). The previously published TaqMan ZIKV 1107 assay was used.^15^ The following controls were included in each RT-qPCR run: six points of ZIKV RNA standard curve (in vitro transcribed oligonucleotide), extraction negative control, extraction positive control, and no template control, all in duplicate.

### RNA recovery estimation.

Ct values and copies/µL values from a duplicate of RT-qPCR for each sample were used to calculate mean Ct values and mean copies/µL values for each extracted sample. Each triplicate of mean copies/µL values per viral load was used to calculate average concentration in spiked matrix for each level of viral load.

To determine expected Ct values and average concentration in spiked reference control, three sets of 10 spiked reference controls samples (triplicates of three viral loads plus one unspiked aliquot) were independently extracted and run on RT-qPCR (one set was run twice). Expected Ct value was calculated as average across all mean Ct values from a duplicate of RT-qPCR for each reference sample of a given viral load. Average concentration in spiked reference control was calculated as average across all mean copies/µL values from a duplicate of RT-qPCR for each reference sample of a given viral load (Supplemental Table 3).

Zika virus RNA recovery was considered acceptable if the sample Ct values were within one Ct unit of the expected Ct value for more than 80% of the replicates of the given sample matrix. If one Ct unit cutoff was not met, two Ct units difference from the expected Ct value for more than 80% of the replicates was considered as the next acceptable criterion. Percent recovery for each level of viral load was calculated as follows:

(average concentration in spiked matrix/average concentration in spiked reference control) × 100%.

### Serum freeze/thaw test.

A known ZIKV-positive serum specimen aliquot^12^ was subjected to five freeze/thaw cycles: an aliquot was taken after each thaw cycle and kept at 4°C in AL buffer/carrier RNA until extraction later on the same day. Each freeze cycle was for 20 minutes at −80°C.

## RESULTS

**Figure 1.**
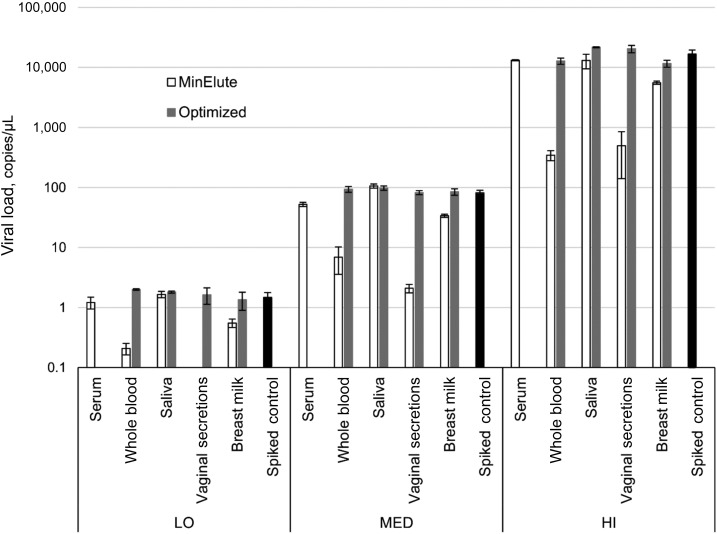
Zika virus RNA recovery from body fluids with the original protocol and after Optimization A. LO = low spiking load; MED = medium spiking load; HI = high spiking load. Bars indicate one standard deviation.

**Figure 2.**
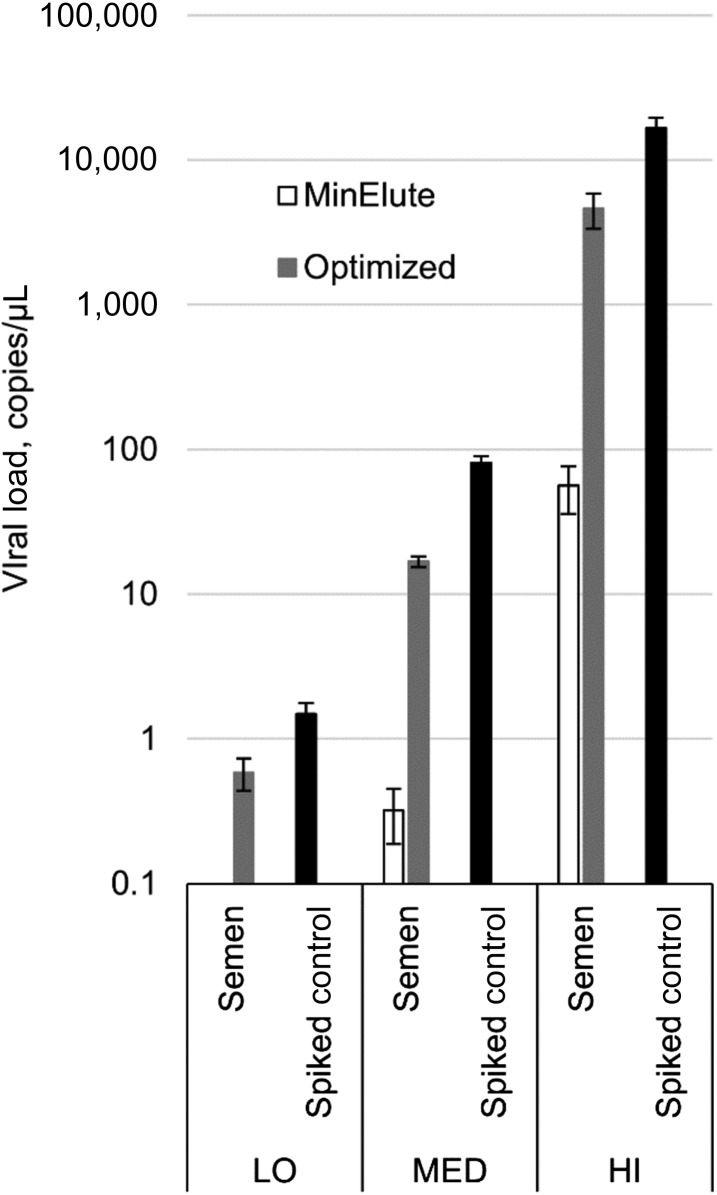
Zika virus RNA recovery from semen specimens with the original protocol and after Optimization B. LO = low spiking load; MED = medium spiking load; HI = high spiking load. Bars indicate one standard deviation.

**Figure 3.**
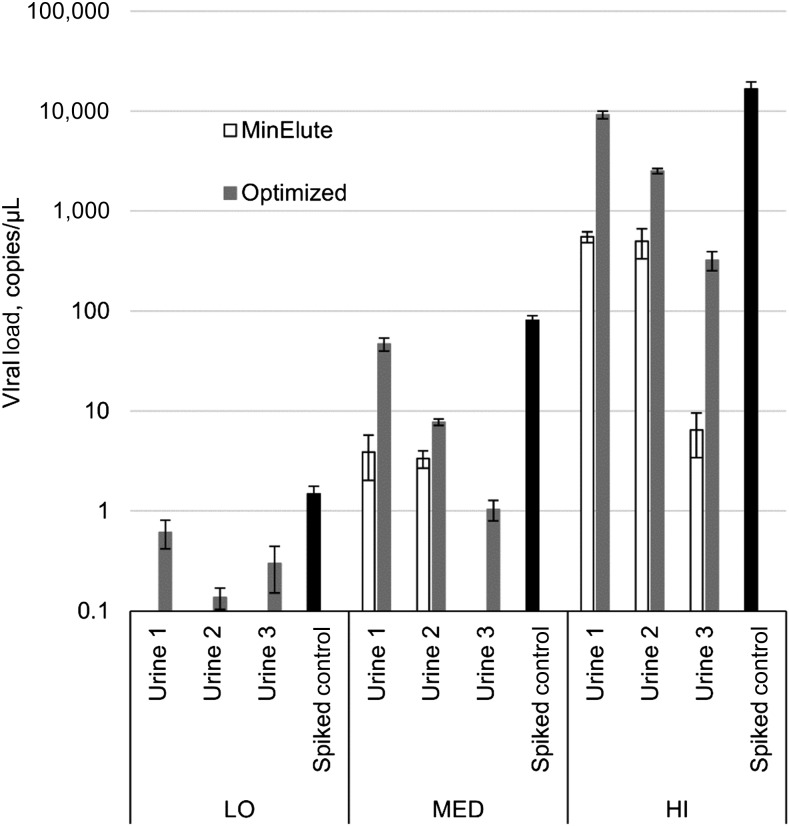
Zika virus RNA recovery from urine specimens of three donors with the original protocol and after Optimization C. LO = low spiking load; MED = medium spiking load; HI = high spiking load. Bars indicate one standard deviation.

Initial testing of the spiked matrices with RNA extracted by the QIAamp MinElute Virus Spin Kit manufacturer’s protocol yielded poor recovery values for all sample types, except for serum and saliva (Figures 1–3; Supplemental Table 4). Because most of the tested matrices contained high amounts of cellular DNA/RNA, the extraction protocol was optimized by addition of 5 minutes incubation at 56°C at the elution step. Slow loading on the extraction column (3,000 rpm) was also added to the protocol to potentially improve recovery of sample nucleic acids. These modifications restored recovery of ZIKV RNA in whole blood, vaginal secretions, and breast milk samples and maintained full recovery in saliva samples (Figure 1; Supplemental Table 4).

The extraction protocol optimized as before did not improve the recovery of ZIKV RNA in urine and semen specimens (Supplemental Tables 1 and 2). A series of alternative extraction protocols were tested for semen specimens and eventually one was selected that yielded 89% of the replicates within two Ct units of the expected Ct values and an average of 29% ZIKV RNA recovery (Figure 2; Supplemental Table 1).

Similarly, a number of additional extraction protocols were tested for urine specimens. The most successful protocol for RNA extraction from urine specimens included addition of UCB and was shown to work with 1-mL aliquots of fresh urine, or frozen “as is.” Spiked urine specimens from a negative control donor were tested and showed 89% of the replicates within two Ct units of the expected Ct values, and an average of 51% ZIKV RNA recovery (Figure 3; Supplemental Table 2). The UCB protocol was applied to spiked urine specimens from two additional negative control donors. In these urine samples, the protocol did not yield expected Ct values, but the results were still the most favorable compared with other available urine preservation methods tested (Figure 3, Supplemental Table 2). However, an abbreviated single spiking load test of two previously available urine specimens, which were stored frozen, showed complete recovery of ZIKV RNA even with the original protocol (Supplemental Table 2). These data can be explained by variation in urine properties from donor to donor.

This optimized urine specimen extraction protocol was applied to specimens serially collected from an acute case of ZIKV infection in a returning traveler.^12^ Urine specimens were stored in 1-mL aliquots at −80°C as is for more than 9 months. In support of the advanced ZIKV RNA detection quality of the UCB extraction protocol, for each time point, Ct values were lower when compared with RNA samples originally extracted from fresh specimens (Table 2).

**Table 2:** Average Ct values of serially collected urine samples from an acute case of Zika virus infection

Extraction method	0 DPO	3 DPO	7 DPO	14 DPO	21 DPO
Fresh urine	37.7	32.1	30.2	36.7	Not detected
Frozen urine, Urine Conditioning Buffer	35.7	31.3	29.9	33.5	37.5

DPO = days post symptoms onset. RNA was extracted either from fresh samples by original protocol, or from stored frozen samples by optimized protocol.

Spiked urine specimens from negative control donors #1 and #3 were tested to compare addition of UCB to a urine specimen stored frozen as is with the addition of UCB to a fresh UR specimen and then freezing and storing the resulting pellet of nucleic acids. There was no difference between these two urine processing methods (Supplemental Table 2, rows 43–46).

To determine if serum specimen freeze/thaw cycles affect Ct values of positive samples in the ZIKV 1107 assay, a known low ZIKV-positive serum specimen was frozen and thawed five times, and aliquots were tested after each thaw cycle. There was no effect of these manipulations on detection of ZIKV RNA by RT-qPCR, *P*-value of the test for trend is 0.317 (Table 3).

**Table 3:** Average Ct values of Zika virus–positive serum sample after serial freeze/thaw treatment

Freeze/Thaw cycle	Average Ct
1	35.07
2	35.10
3	35.27
4	34.42
5	34.54

## DISCUSSION

Using QIAamp MinElute Virus Spin Kit as basic RNA extraction method, we evaluated protocol variations to optimize detection of ZIKV in various body fluids using RT-qPCR, as means of improving the analytical sensitivity of detection. We have shown that several adaptations of the extraction protocol, in combination with the previously published TaqMan ZIKV 1107 RT-qPCR assay, provide a reliable method for detection of ZIKV RNA in a variety of specimens. Serum and saliva swab specimens had optimal recovery of ZIKV RNA with the original manufacturer’s protocol and did not require additional extraction adaptations.

Whole blood, vaginal secretions swabs, and breast milk specimens required extended incubation at elevated temperature at the elution step for optimal recovery of viral RNA (Figure 1; Supplemental Table 4). The adjustments also included slow load of the sample onto the column, but the main effect of the RNA recovery restoration was achieved because of the modification of the elution step. The specimen types that benefited from this Optimization A protocol have significant cellular content, and it was assumed that more stringent elution conditions would be required to release high amount of cellular nucleic acids (along with ZIKV RNA) from the column filter. Despite excellent performance of the original protocol for saliva specimens, optimized extraction protocol would be recommended for this matrix because of potential variation in the amount of cellular DNA/RNA content.

Semen specimens required the use of high volume of RLT Plus buffer and RNeasy Mini Spin Columns (Figure 2; Supplemental Table 4). Of the several tested methods, these two simple modifications proved to be the best technique of managing extremely high DNA content in semen specimens. Although we could not reach full recovery of ZIKV RNA from this matrix, our result (29%) was very similar to that of a recently published study from the Centers for Disease Control and Prevention (CDC) group in Puerto Rico.^16^ The investigators showed an average of 31% ZIKV RNA recovery from semen specimens using Trioplex Real-time RT-PCR Assay testing platform, with magnetic beads–based automated sample RNA extraction.

Urine specimens are infamous for the difficulties associated with RNA extraction from this specimen matrix.^17,18^ We applied a number of modifications to the original protocol, and had the most success with addition of UCB from Zymo Research. We were able to reach a cut-off of two Ct units difference relative to the spiked buffer reference (Figure 3; Supplemental Table 4). However, because the extractions with this protocol are performed from 1 mL of urine sample, compared with 0.2 mL in the original protocol, expected Ct values are decreased by 2.3 units. In that regard, although average recovery was 51%, Ct values of all replicates were actually lower than expected Ct values when extracted from 0.2 mL of urine sample, suggesting that this extraction protocol offers a more sensitive test. We also showed that addition of UCB could be performed before or after freezing the specimen for storage, with the same effect on RNA recovery. However, in our experiment, specimens were kept as is at −80°C only for 1 day. Therefore, storing of urine specimens preserved with UCB right after the sample collection should be preferable because of potential decrease in recovery of low levels of ZIKV RNA when stored as is for prolonged time, as it was shown in a recent study.^18^ On the other hand, we used the UCB method with archived ZIKV-positive urine specimens (stored for more than 9 months at −80°C without any preservatives), and were able to achieve better Ct values when compared with extractions performed right after specimen collection by the standard protocol (Table 1). This variability in success of RNA extraction from urine specimens of different subjects was also evident in reduced efficiency of UCB for spiked specimens of two additional negative donors that we tested. Further research on consistent methods of RNA extraction from urine specimens is required.

Our long-term goal is to apply these methods for optimal RNA recovery to determine shedding time in different body fluids among a cohort of ZIKV-infected patients to identify opportunities for a longer window of time to diagnose infection. In addition to specimen type and timeframe recommendations for viral RNA tests in laboratory diagnosis of acute infections, cerebrospinal fluid (CSF) and amniotic fluid specimens are also acceptable for testing, but must have a paired serum specimen as recovery of RNA from these alternative specimens can be highly variable. Zika virus RNA has been detected in other body fluids and, for some matrices, far past the acute stage of the disease. This RNA persistence was shown in clinical studies and in animal models.^5,12,16,19–25^ Several case reports and case series documented ZIKV RNA detection in whole blood up to 81 days post symptoms onset (DPO),^12,20^ in saliva up to 60 DPO,^12,16,26^ in vaginal secretions up to 14 DPO,^12,27,28^ and in breast milk up to 8 days post delivery.^29^ A recent Puerto Rican prospective cohort study of 150 symptomatic participants who tested positive by RT-PCR at presentation showed the following medians and 95^th^ percentiles for the time until loss of ZIKV RNA detection: 14 and 54 days, respectively, in serum; 8 and 39 days in urine, and 34 and 81 days in semen.^16^ In animal models, ZIKV RNA was shown to persist for several weeks after infection in saliva, lymph nodes, and colorectal tissue in cynomolgus and rhesus macaques.^21–25^

Studies that generated aforementioned results on prolonged detection of ZIKV RNA in tissues and body fluids mostly used the generalized RNA extraction protocols provided by the extraction kit manufacturers, without confirming (or at least without reporting) the level of viral RNA recovery for a given specimen type and a given RNA extraction method. One exception is the Puerto Rican cohort study where ZIKV RNA detection was performed by Trioplex Real-time RT-PCR Assay, which is validated by CDC for ZIKV for use with serum; and with urine, CSF, and amniotic fluid only alongside the patient-matched serum specimen.^16^ However, even the same study showed only about 30% recovery of ZIKV RNA from semen specimens with their assay. Semen, as well as urine and whole blood, are known for their complex biochemical and cellular composition that present challenges for complete and PCR inhibitor-free extractions of RNA or DNA.^17,18,30–35^ Several studies have compared the effectiveness of different nucleic acid extraction methods from these body fluids,^17,18,30,31,36^ rarely showing actual viral RNA recovery compared with buffer control.^37^

A main limitation to our study was that we have not tested stability of ZIKV in various body fluids as applied to time from collection to time of addition of lysis buffer and following processing of the sample. Our study was focused on effect of a matrix in terms of its composition on RNA extraction and coupled RT-qPCR. Hence, the specimens were first lysed, and then spiked, to differentiate form any effect of processes that might happen in a clinical specimen in its natural state. We were also limited in testing variations of RNA extraction as applied to a single commercially available kit. Although this QIAamp MinElute Virus Spin Kit is widely used in viral research, there are many other RNA extraction kits on the market, including automation equipment/kits used in clinical diagnostic laboratories. Our results might provide guidance in optimizing RNA recovery when using these alternative methods. Finally, our study design was limited in testing whole blood specimens that lack most of the plasma component. Different amounts of plasma in separated versus complete whole blood specimens might change the performance of the RNA extraction kit. However, we would expect the effect to be minimal as plasma is one of the specimen types for which the QIAamp MinElute Virus Spin Kit was developed.^14^ Another concern of adding a step of plasma separation in the whole blood testing procedure is an increased risk of contamination of the sample. This risk increase can be alleviated by strict adherence to high standards of laboratory practices.

Optimal methods for ZIKV detection in various body fluids will allow accurate monitoring of viral RNA presence during the disease progression in the future studies aimed to improving our understanding of natural history of ZIKV infection and duration of viral persistence in various body fluids. Optimized methods can potentially be successfully used with other arboviruses, as improved diagnostics are crucial to timely detection and response to emerging infections such as ZIKV.

## Supplementary Material

Supplemental figure
