# Supplementary material for: Optimizing PCR Detection of Zika Virus from Various Body Fluids
Source: Am J Trop Med Hyg. 2018 Dec 17;100(2):427–33. doi: 10.4269/ajtmh.18-0755 (PMC6367632; doi:10.4269/ajtmh.18-0755)
Supplement: Supplementary file 3 [file tpmd180755.SD3.pdf]

SUPPLEMENTAL TABLE 3. Individual and average (expected) Ct values and respective concentrations of ZIKV RNA generated by repeated testing of the spiked reference control replicates

Replicates	Set 1		Set 2		Set 3, run 1		Set 3, run 2		Average		SD	
	Ct	cp/µl	Ct	cp/µl	Ct	cp/µl	Ct	cp/µl	Ct	cp/µl	Ct	cp/µl
LO 1	33.8	1.8	33.9	1.8	33.3	1.5	33.9	1.7	33.9	1.5	0.4	0.3
LO 2	34.9	0.8	33.9	1.7	33.7	1.1	33.8	1.8				
LO 3	34.1	1.4	34.2	1.4	33.3	1.5	34.2	1.4				
MED 1	28.5	65.0	28.2	91.8	27.7	78.9	28.1	94.3	28.2	81.1	0.3	8.8
MED 2	28.3	77.4	28.2	92.6	27.6	79.3	28.3	84.4				
MED 3	28.4	71.3	28.4	83.8	27.7	78.0	28.4	76.7				
HI 1	21.1	11127.5	20.8	14988.9	20.7	17790.7	20.7	15865.1	20.6	16665.9	0.2	2869.9
HI 2	20.8	13303.1	20.5	18488.1	20.7	17154.8	20.7	15506.6				
HI 3	20.1	21133.0	20.3	20277.0	20.6	18833.2	20.7	15523.1				

SD = standard deviation; cp/µl = copies per microliter; LO = low spiking load; MED = medium spiking load; HI = high spiking load.
