# Supplementary material for: Optimizing PCR Detection of Zika Virus from Various Body Fluids
Source: Am J Trop Med Hyg. 2018 Dec 17;100(2):427–33. doi: 10.4269/ajtmh.18-0755 (PMC6367632; doi:10.4269/ajtmh.18-0755)
Supplement: Supplementary file 4 [file tpmd180755.SD4.pdf]

SUPPLEMENTAL TABLE 4. Individual Ct values and percent recovery of ZIKV RNA in various body fluid specimens using original and optimized extraction protocols

	Rep 1 Ct	Rep 2 Ct	Rep 3 Ct	Expected Ct	#Rep within 1 Ct	% recovery	Average % recovery
SR LO	34.8	34.1	34.4	33.9	3/3	82	75
SR MED	28.2	28.4	28.1	28.2	3/3	64	
SR HI	21.1	21.1	21.1	20.6	3/3	79	
WB LO	37.4	38.0	38.0	33.9	0/3	14	8
WB MED	32.2	31.7	33.3	28.2	0/3	9	
WB HI	26.3	26.8	26.2	20.6	0/3	2	
WB optimized LO	33.9	33.9	33.8	33.9	3/3	135	109
WB optimized MED	28.6	28.4	28.7	28.2	3/3	115	
WB optimized HI	21.3	21.0	21.2	20.6	3/3	77	
SS LO	33.7	33.8	33.5	33.9	3/3	112	107
SS MED	27.7	27.4	27.6	28.2	3/3	131	
SS HI	20.3	21.6	20.4	20.6	3/3	78	
SS optimized LO	34.0	34.1	34.0	33.9	3/3	122	124
SS optimized MED	28.7	28.5	28.4	28.2	3/3	121	
SS optimized HI	20.4	20.4	20.4	20.6	3/3	130	
VS LO	ND	ND	DC	33.9	0/3	0	2
VS MED	33.2	33.1	33.6	28.2	0/3	3	
VS HI	25.2	24.6	27.4	20.6	0/3	3	
VS optimized LO	34.6	34.3	33.7	33.9	3/3	110	111
VS optimized MED	28.5	28.3	28.5	28.2	3/3	101	
VS optimized HI	20.5	20.4	20.1	20.6	3/3	123	
BM LO	35.7	35.5	36.0	33.9	0/3	37	38
BM MED	29.6	29.4	29.6	28.2	0/3	42	
BM HI	22.0	22.0	21.8	20.6	0/3	33	
BM optimized LO	34.6	33.8	34.7	33.9	3/3	91	88
BM optimized MED	28.4	28.1	28.0	28.2	3/3	104	
BM optimized HI	21.1	20.8	20.7	20.6	3/3	70	
SM LO	40.6	ND	ND	33.9	0/3	0	0
SM MED	36.5	35.7	36.9	28.2	0/3	0	
SM HI	28.2	29.9	29.1	20.6	0/3	0	
SM optimized LO	34.8	35.0	34.3	33.9	3/3*	40	29
SM optimized MED	30.0	29.8	29.8	28.2	3/3*	21	
SM optimized HI	22.4	22.4	23.2	20.6	2/3*	28	
UR1 LO	ND	DC	DC	33.9	0/3	0	3
UR1 MED	32.5	32.6	34.1	28.2	0/3	5	
UR1 HI	25.4	26.1	25.7	20.6	0/3	3	
UR1 optimized LO	32.8	33.3	33.7	31.6**	2/3*	41	51

UR1 optimized MED	27.2	26.7	27.0	25.9**	3/3*	58	
UR1 optimized HI	19.4	19.5	19.2	18.3**	3/3*	55	
UR2 LO	DC	ND	ND	33.9	0/3	NC	NC
UR2 MED	33.9	33.4	34.0	28.2	0/3	NC	
UR2 HI	26.0	27.0	26.3	20.6	0/3	NC	
UR2 optimized LO	35.7	35.1	35.1	31.6**	0/3*	NC	NC
UR2 optimized MED	29.4	29.5	29.3	25.9**	0/3*	NC	
UR2 optimized HI	21.0	21.1	21.0	18.3**	0/3*	NC	
UR3 LO	ND	ND	ND	33.9	0/3	NC	NC
UR3 MED	38.1	ND	DC	28.2	0/3	NC	
UR3 HI	31.4	29.9	30.0	20.6	0/3	NC	
UR3 optimized LO	38.5	DC	37.4	31.6**	0/3*	NC	NC
UR3 optimized MED	32.3	32.4	33.0	25.9**	0/3*	NC	
UR3 optimized HI	25.1	24.5	24.9	18.3**	0/3*	NC	

* = number of biological replicates within 2 Ct units from expected Ct; ** = expected Ct decreased by 2.3 due to 5-fold higher volume of spiked sample (1 ml vs. 0.2 ml); Rep = biological replicate; SR = serum; WB = whole blood; SS = saliva swabs; VS = vaginal secretions swabs; BM = breast milk; SM = semen; UR = urine; LO = low spiking load; MED = medium spiking load; HI = high spiking load; ND = not determined; DC = discordant; NC = not calculated.
